# Supplementary material for: Cas4/1 dual nuclease activities enable prespacer maturation and directional integration in a type I-G CRISPR-Cas system
Source: bioRxiv. 2023 Jun 5:2023.06.05.543779. Preprint. [Version 1] doi: 10.1101/2023.06.05.543779 (PMC10274657; doi:10.1101/2023.06.05.543779)
Supplement: Supplement 1 [file media-1.pdf]

Supplementary Information

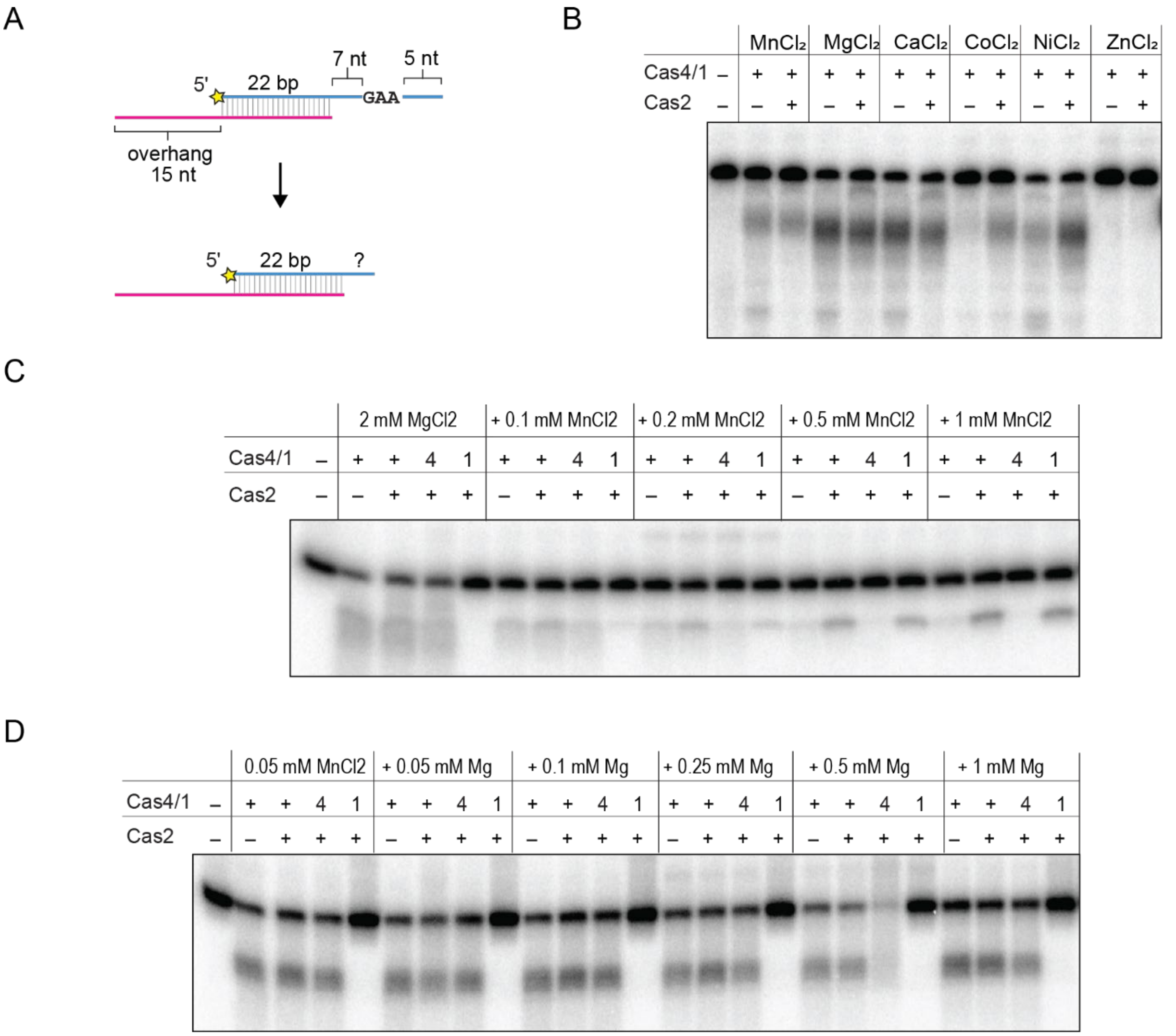

Supplementary Figure 1. Cas4/1 nuclease activity in the presence of various metal ion cofactors

A, Schematic of cleavage assay for a PAM/NoPAM substrate with the PAM strand radiolabeled**.** Radioactive label is indicated with a yellow star. **B,** Denaturing polyacrylamide gel showing cleavage assay with substrate shown in (A) in the presence of various metal ion cofactors. Metal ions were used at a final concentration of 2 mM. **C,** Cleavage assay for substrate shown in (A) with constant concentration of 2 mM $MgCl_2$ and increasing concentrations of $MnCl_2$ in the reaction. **D,** Cleavage assay for substrate shown in A with constant concentration of 0.05 mM $MnCl_2$ and increasing concentrations of $MgCl_2$ in the reaction.

Supplementary Table 1. Primer used in this study

**Name**	**Sequence (5′ to 3′)**	**Description**
1	GTGGCGTACAAGAAGGGCTATGTACCTG	Forward for MbCas4/1 E101A
2	GGTACGGCTTTGCCGTTACTTTC	Reverse for MbCas4/1 E101A
3	ATTGCGGGAACTGCAGCCCAAATTTATTTTTCC	Forward for MbCas4/1 E375A
4	CCCCAATAACTGACCGATCTCC	Reverse for MbCas4/1 E375A
5	GATCCGAGACGAGACAGTACACAACATGTGAAT<br>GCCCATACTGTCCCTGGCTTCAATTCTGCCACAAC<br>CTTTCGGTTATGGAAACGGCGC	Oligo 1 for MbCRISPR assembly in pUC19 – complementary to oligo 4
6	TAAGATCTTCACGTCTATTAGCTACGGAATTCACT<br>TTGTTTTCGAGAGATCATTGAATTGAATTCTTTTC<br>ATGGATTATAAAACTAGCAT	Oligo 2 for MbCRISPR assembly in pUC19 – complementary to oligo 5
7	ATTTATCTCAATTATAAAAGCTGAAGCTTCTCGA<br>GAGCCTTCAGCAGTTTTTAGGGTTCATAAGCTCTC<br>GAAAACG	Oligo 3 for MbCRISPR assembly in pUC19 – complementary to oligo 6
8	AGGTTGTGGCAGAATTGAAGCCAGGGACAGTATG<br>GGCATTCACATGTTGTGTACTGTCTCGTCTCG	Oligo 4 for MbCRISPR assembly in pUC19
9	GAAAAGAATTCAATTCAATGATCTCTCGAAAACA<br>AAGTGAATTCCGTAGCTAATAGACGTGAAGATCT<br>TAGCGCCGTTTCCATAACCGAA	Oligo 5 for MbCRISPR assembly in pUC19
10	AATTCGTTTTCGAGAGCTTATGAACCCTAAAAAC<br>TGCTGAAGGCTCTCGAGAAGCTTCAGCTTTTATA<br>ATTGAGATAAATATGCTAGTTTTATAATCCAT	Oligo 6 for MbCRISPR assembly in pUC19
11	GTCTCGTGGGCTCGGAGATGTGTATAAGAGACAG<br>CGTAGCTGAGGACCACCAGTAC	Prespacer top/PAM strand with Nextera adaptor – for L1 or S1
12	GTCTCGTGGGCTCGGAGATGTGTATAAGAGACAG<br>GTACTGGTGGTCCTCAGCTACG	Prespacer bottom/NoPAM strand with Nextera adaptor – for L2 or S2
13	TCGTCGGCAGCGTCAGATGTGTATAAGAGACAGG<br>AGACGAGACAGTACACAACATGTG	MbCRISPR backbone with Nextera adaptor – for L1 and L2
14	TCGTCGGCAGCGTCAGATGTGTATAAGAGACAGC<br>AAAGTGAATTCCGTAGCTAATAGACG	MbCRISPR leader with Nextera adaptor – for S1 and S2

Supplementary Table 2. Oligonucleotides used in this study

Name	Sequence (5′ to 3′)	Description
1	CGTAGCTGAGGACCACCAGTACTTTTTTTGA ATTTTT	PAM strand for PAM/NoPAM or PAM/Proc prespacer (PAM 7 nt away from duplex)
2	GTACTGGTGGTCCTCAGCTACGTTTTTTTTTT TTTTT	NoPAM strand for PAM/NoPAM or prespacer
3	CGTAGCTGAGGACCACCAGTACTTTTTTT	Top strand for Proc/Proc prespacer
4	GTACTGGTGGTCCTCAGCTACGTTTTTTT	Bottom strand for Proc/Proc prespacer
5	CGTAGCTGAGGACCACCAGTACTTTGAATTT TTTTTT	PAM strand with PAM 3 nt away from duplex (Fig. 2)
6	CGTAGCTGAGGACCACCAGTACTTTTTGAAT TTTTTT	PAM strand with PAM 5 nt away from duplex (Fig. 2)
7	CGTAGCTGAGGACCACCAGTACTTTTTTTTTG AATTT	PAM strand with PAM 9 nt away from duplex (Fig. 2)
8	AGGACAACGTTACGGACGGCACAGCCTTTTT GAATT	PAM strand of prespacer for prespacer vs HSI cleavage assay (Fig. 3)
9	CCCTGTGCCGTCCGTAACGTTGTCGATTTTT	Proc strand of prespacer for prespacer vs HSI cleavage assay (Fig. 3)
10	CCCTGTGCCGTCCGTAACGTTGTCGATTTTTG TTTCCATAACCGAAAGGTTGTGGCAGAATTG AAGCGGCTTC	HSI substrate top strand with processed strand integrated at leader-side with repeat and spacer (Fig. 3)
11	GAAGCCGCTTCAATTCTGCCACAACCTTTCG GTTATGGAAACGGCGCTAAGATCTTTTGAT CTTAGCGCC	HSI substrate bottom strand with leader hairpin, repeat and spacer (Fig. 3)
12	AGGACAACGTTACGGACGGCACAGCCT	3 nt overhang length strand for spacer side integration assay (Fig. 3)
13	AGGACAACGTTACGGACGGCACAGCCTT	4 nt overhang length strand for spacer side integration assay (Fig. 3)
15	AGGACAACGTTACGGACGGCACAGCCTTT	6 nt overhang length strand for spacer side integration assay (Fig. 3)
15	AGGACAACGTTACGGACGGCACAGCCTTTT	6 nt overhang length strand for spacer side integration assay (Fig. 3)
16	AGGACAACGTTACGGACGGCACAGCCTTTTT	7 nt overhang length strand for spacer side integration assay (Fig. 3)
17	AGGACAACGTTACGGACGGCACAGCCTTTTT	8 nt overhang length strand for spacer side integration assay (Fig. 3)
